# eIF4A Inhibition Allows Translational Regulation of mRNAs Encoding Proteins Involved in Alzheimer's Disease

**DOI:** 10.1371/journal.pone.0013030

**Published:** 2010-09-28

**Authors:** Andrew Bottley, Nicola M. Phillips, Thomas E. Webb, Anne E. Willis, Keith A. Spriggs

**Affiliations:** School of Pharmacy, University of Nottingham, Nottingham, United Kingdom; Case Western Reserve University, United States of America

## Abstract

Alzheimer's disease (AD) is the main cause of dementia in our increasingly aging population. The debilitating cognitive and behavioral symptoms characteristic of AD make it an extremely distressing illness for patients and carers. Although drugs have been developed to treat AD symptoms and to slow disease progression, there is currently no cure. The incidence of AD is predicted to increase to over one hundred million by 2050, placing a heavy burden on communities and economies, and making the development of effective therapies an urgent priority. Two proteins are thought to have major contributory roles in AD: the microtubule associated protein tau, also known as MAPT; and the amyloid-beta peptide (A-beta), a cleavage product of amyloid precursor protein (APP). Oxidative stress is also implicated in AD pathology from an early stage. By targeting eIF4A, an RNA helicase involved in translation initiation, the synthesis of APP and tau, but not neuroprotective proteins, can be simultaneously and specifically reduced, representing a novel avenue for AD intervention. We also show that protection from oxidative stress is increased upon eIF4A inhibition. We demonstrate that the reduction of these proteins is not due to changes in mRNA levels or increased protein degradation, but is a consequence of translational repression conferred by inhibition of the helicase activity of eIF4A. Inhibition of eIF4A selectively and simultaneously modulates the synthesis of proteins involved in Alzheimer's disease: reducing A-beta and tau synthesis, while increasing proteins predicted to be neuroprotective.

## Introduction

Alzheimer's disease (AD) is the main cause of dementia in our aging population, and currently there is no effective treatment. At the cellular and molecular level, AD is characterized by the presence of extracellular plaques of the peptide amyloid-beta (Aβ), and also by intracellular neurofibrillary tangles of tau protein (reviewed in [1]). The precise molecular basis of AD has been the subject of an enormous amount of research, and the details are still the subject of some disagreement. Nevertheless, it is clear that amyloid precursor protein (APP, NM_000484) and tau (NM_016835) play important roles in disease progression, and both have been identified as promising therapeutic targets [2], [3], [4], [5], [6], [7]. Reducing the levels of APP and tau has been shown to slow disease progress in animal models, and current therapies target disease models based on these two proteins, albeit with incomplete success. For example, even a modest reduction of soluble Aβ has been shown to have a dramatic effect on amyloid plaque formation in animal models [8], and reduction of tau protein levels ameliorates the neurotoxic effects of Aβ in mice overexpressing APP [9]. Oxidative stress is also implicated as an important factor in AD progression, and increasing the levels of proteins involved in reducing oxidative stress are predicted to impact beneficially on AD symptoms (reviewed in [10], [11]). Oxidative stress is likely an early event in AD progression, and has been shown to increase Aβ formation by increasing APP levels and processing [12], [13]. Aβ itself has oxidant properties, leading to a positive feedback loop and further increase in Aβ levels [14], [15]. In addition to increasing the likelihood of plaque formation, increased Aβ levels also lead to hyperphosphorylation of tau protein, an increase in neurofibrillary tangle formation, and consequent further oxidative stress [14]. Markers of oxidative stress, including modification of DNA, RNA, lipids and proteins, are increased in brains of AD patients, and in animal models [11]. In contrast to other cell types, the high metabolic rates and levels of pro-oxidants, coupled with less efficient anti-oxidant responses, makes cells of the CNS particularly prone to oxidative stress [10]. In mouse models incorporating both Aβ and tau pathologies, Aβ and tau proteins were found to act synergistically to inhibit mitochondrial oxidative phosphorylation, and increase cellular levels of reactive oxygen species [16].

The regulation of protein synthesis at the level of translation is particularly important in neuronal cells for a number of reasons. Firstly, neurons tend to be metabolically very active, placing heavy demands on the protein synthesis machinery. Secondly, the lengths of many of the cells of the CNS means that controlling the rates of translation of localized mRNA pools will likely offer faster and more flexible control of gene expression than changes in the levels of transcription, which would necessarily involve transport of either newly synthesized mRNA or protein.

The most abundant eukaryotic translation initiation factor is eIF4A, an RNA helicase which facilitates translation initiation by unwinding otherwise inhibitory mRNA structure. The 5′ untranslated regions (5′ UTRs) of APP and tau mRNAs are known to be long and structured, and hence represent a barrier to the initiating ribosome during protein synthesis. Additionally, both APP and tau mRNAs are able to initiate translation in a cap-independent manner via internal ribosome entry sites (IRESes) [17], [18]. We have recently shown that eIF4A is required for a number of mammalian IRESes [19]. We therefore propose that inhibiting eIF4A will reduce the efficiency of APP and tau protein synthesis, but not that of housekeeping proteins. Interestingly, it has been reported that decreasing the stability of RNA structures within portions of the APP 5′ UTR is correlated with increased expression [20]. We have used hippuristanol, a potent and specific inhibitor of eIF4A, to test this model. Hippuristanol, a polyoxygenated steroid isolated from the Gorgonian *Isis hippuris* has been shown to be a potent and specific inhibitor of the helicase, RNA binding and ATP hydrolysis properties of eIF4A [21]. APP, tau and Aβ levels were found to be reduced in hippuristanol treated SH-SY5Y cells, whereas proteins involved in defence from oxidative stress are increased (e.g. SOD1, TXN and NDUFB2). We show that these changes are due to differences in *de novo* protein synthesis rates mediated via the 5′ UTRs, and not to variation in transcription or protein turnover, and that cell viability under oxidative insult is increased.

## Results

### Polysomal redistribution of mRNAs following hippuristanol treatment

**Figure 1:**
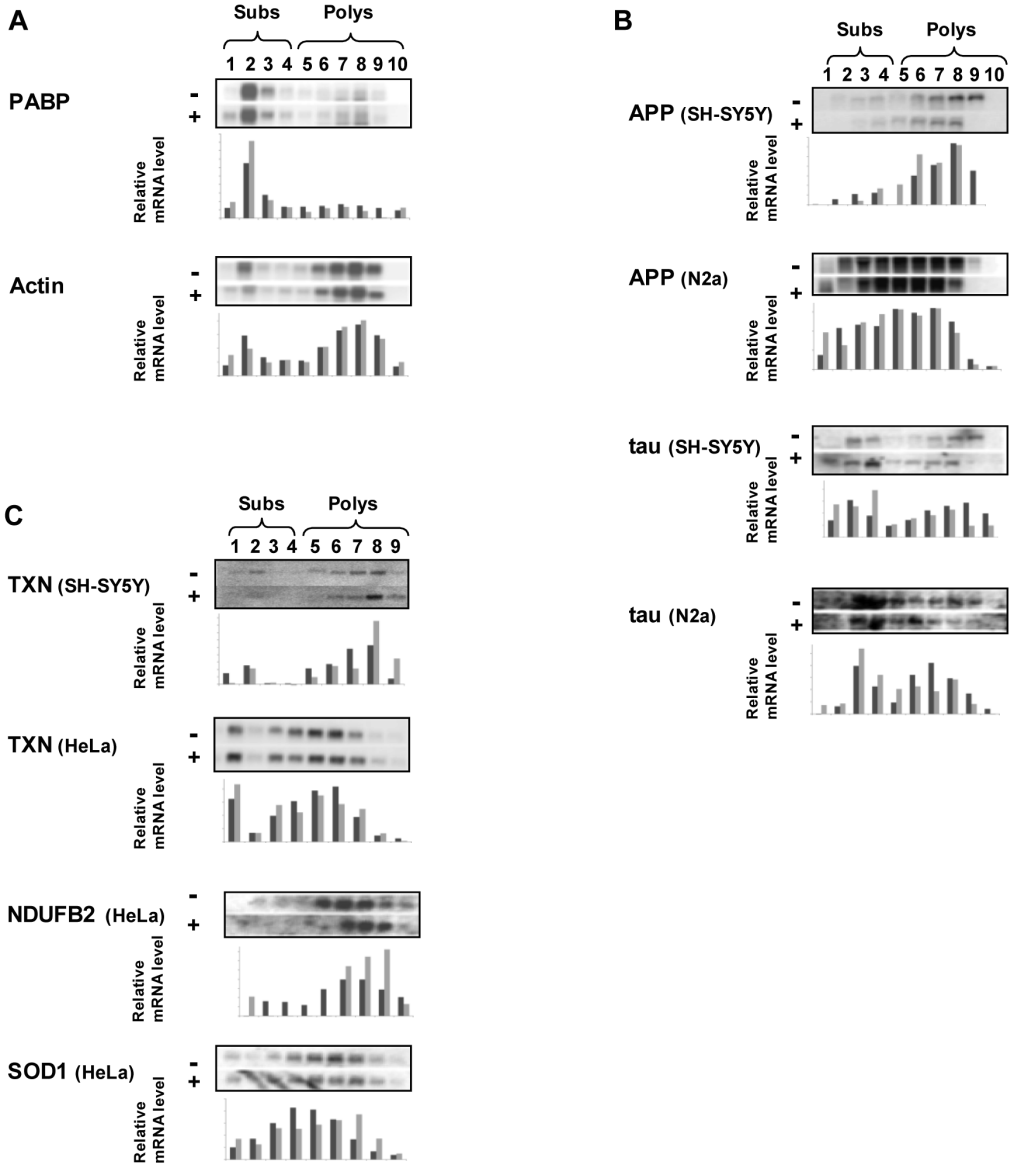
eIF4A inhibition changes the polysomal association of certain mRNAs. Cultured cells (SH-SY5Y, N2a or HeLa) were treated with 10 µM hippuristanol (+) or DMSO control (−) for 10 minutes, lysed and mRNA fractionated on a 10%–50% sucrose gradient. OD260nm absorbance was determined with simultaneous 1 ml fraction collection to determine subpolysomal and polysomal fractions (fraction number and subpolysomal or polysomal association indicated). Northern analysis was performed to determine the positions of mRNAs for: **A** Actin and PABP which do not change in polysome associations; **B** APP and tau which become less polysomally associated; **C** TXN, NDUFB2 and SOD1 which become more polysomally associated. Relative levels of RNA are indicated by dark grey (DMSO control) and light grey (10 µM hippuristanol) bars beneath each fraction, as percentages of the total amount RNA in each gradient.

To test the effects of eIF4A inhibition on the translation of proteins involved in AD, hippuristanol was used to treat cultured mammalian cells for ten minutes (HeLa, N2a and SH-SY5Y), and the polysomal associations of APP, tau, TXN (thioredoxin; NM_003329), SOD1 (superoxide dismutase 1; NM_000454), NDUFB2 (NADH dehydrogenase (ubiquinone) 1 beta subcomplex, 2; NM_004546), β-actin (NM_001101) and PABP (poly-A binding protein; NM_002568) were each determined in treated and control cells using sucrose density gradient centrifugation followed by northern analysis (figure 1). Sucrose density gradient centrifugation separates mRNAs according to their ribosomal load, with actively translated (polysomal) mRNAs partitioning towards the bottom of the gradient due to their increased density, whereas subpolysomal mRNAs are less dense and partition towards the top of the gradient. The polysomal distributions of β-actin and PABP were unchanged following hippuristanol treatment (figure 1A), however, it is clear that the mRNAs encoding APP and tau shift subpolysomally following hippuristanol treatment indicating a reduction in translation of these transcripts (figure 1B). In contrast, mRNAs encoding SOD1, TXN, and NDUFB2 (which have been proposed to have neuroprotective roles [22], [23], [24] increase in polysomal association (figure 1C).

### Changes in AD associated protein levels in hippuristanol treated cells

**Figure 2:**
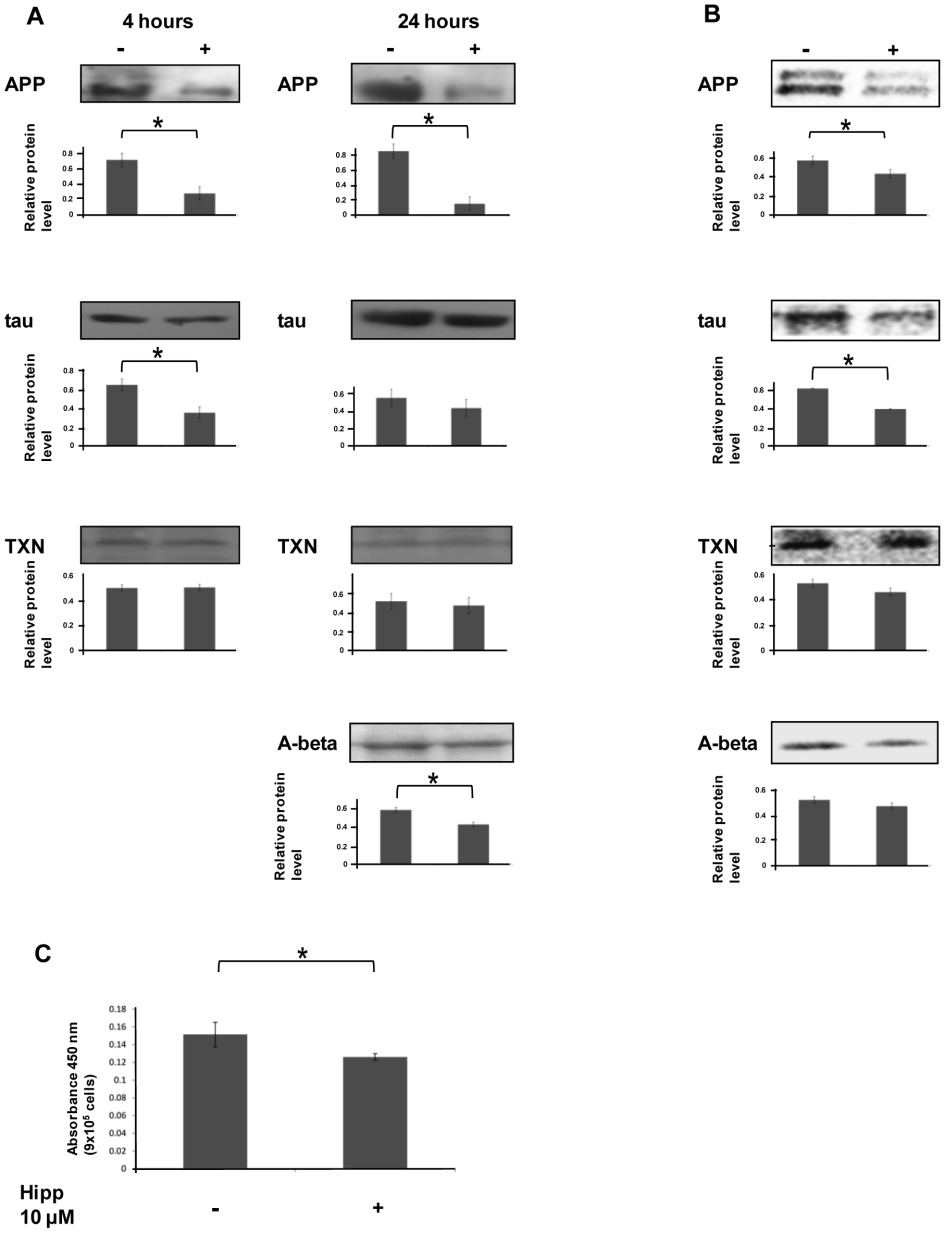
Expression of proteins associated with AD is altered by eIF4A inhibition. **A** Western analysis was performed on lysates from SH-SY5Y cells treated with 10 µM hippuristanol (+) or DMSO control (−) for 4 or 24 hours with antibodies specific to APP, TAU and TXN (bands of around 70 kDa, 60 kDa and 12–14 kDa respectively). The 70 kDa band was detected by the APP specific antibody representing a cleavage product specific to K+ isoforms [53]. Secreted A-beta was measured by western analysis of cell culture media. The 56 kDa product is interpreted as an aggregate of A-beta, as has previously been reported for western analysis of brain tissue [51]. Protein levels were normalized to actin, and mean relative proportions of protein with standard errors are indicated beneath each gel image. Significant differences (p≤0.05 by t-test) are indicated by asterisks. **B**. Immunoprecipitation following ^35^S-methionine labeling demonstrates a reduction in novel synthesis of APP, TAU but not TXN when SH-SY5Y cells are treated with 10 µM hippuristanol. Secreted A-beta levels are also reduced, as determined by immunoprecipitation from cell culture medium. Mean relative proportions of protein with standard errors are indicated beneath each gel image. Significant differences (p≤0.05 by t-test) are indicated by asterisks. **C**. Secreted A-beta levels in the medium of 9×10^5^ SH-SY5Y cells were assayed by ELISA 24 hours following treatment with 10 µM hippuristanol (+), demonstrating a modest but reproducible decrease in comparison to control cells (−). Means and standard errors of 6 replicates are shown p = 0.05.

To determine whether these changes in polysomal association result in alterations to the cellular concentrations of proteins involved in AD, western analysis was performed using antibodies specific to APP, tau, TXN, Aβ and β-actin (figure 2). APP concentrations in SH-SY5Y cells were markedly reduced at 4 and 24 hours following 10 µM hippuristanol treatment, and tau protein levels showed a reduction at 4 hours followed by a partial recovery by 24 hours following treatment (figure 2A). Conversely, TXN levels increased slightly 4 hours following hippuristanol treatment and returned to control levels within 24 hours. APP is cleaved to generate the Aβ peptide, and it is the aberrant aggregation of this peptide into extracellular plaques that is characteristic of AD pathology. Western blotting of concentrated media shows a reduction of secreted Aβ peptide 24 hours following hippuristanol treatment of SH-SY5Y cells (figure 2A). Treatment with 20 µM and 30 µM hippuristanol increases this reduction of APP, tau and Aβ levels (data not shown).

### Hippuristanol reduces APP and tau protein synthesis

To establish whether the observed changes in APP, tau and TXN protein levels in hippuristanol treated cells were due to changes in new protein synthesis rather than turnover, synthesis rates were measured by immunoprecipitation following ^35^S-methionine labeling of SH-SY5Y cells (figure 2B). Levels of APP and tau synthesis were markedly reduced following hippuristanol treatment, whereas TXN synthesis was unaffected. To determine the effects of hippuristanol on extracellular levels of Aβ, immunoprecipitation from the media of hippuristanol treated and control SH-SY5Y cells was performed using an antibody specific to Aβ following ^35^S-methionine labeling. A decrease in extracellular Aβ was observed by western blotting of the media of treated cells (figure 2B) and also by ELISA (figure 2C).

### Changes in mRNA levels are not responsible for the observed changes in protein levels

**Figure 3:**
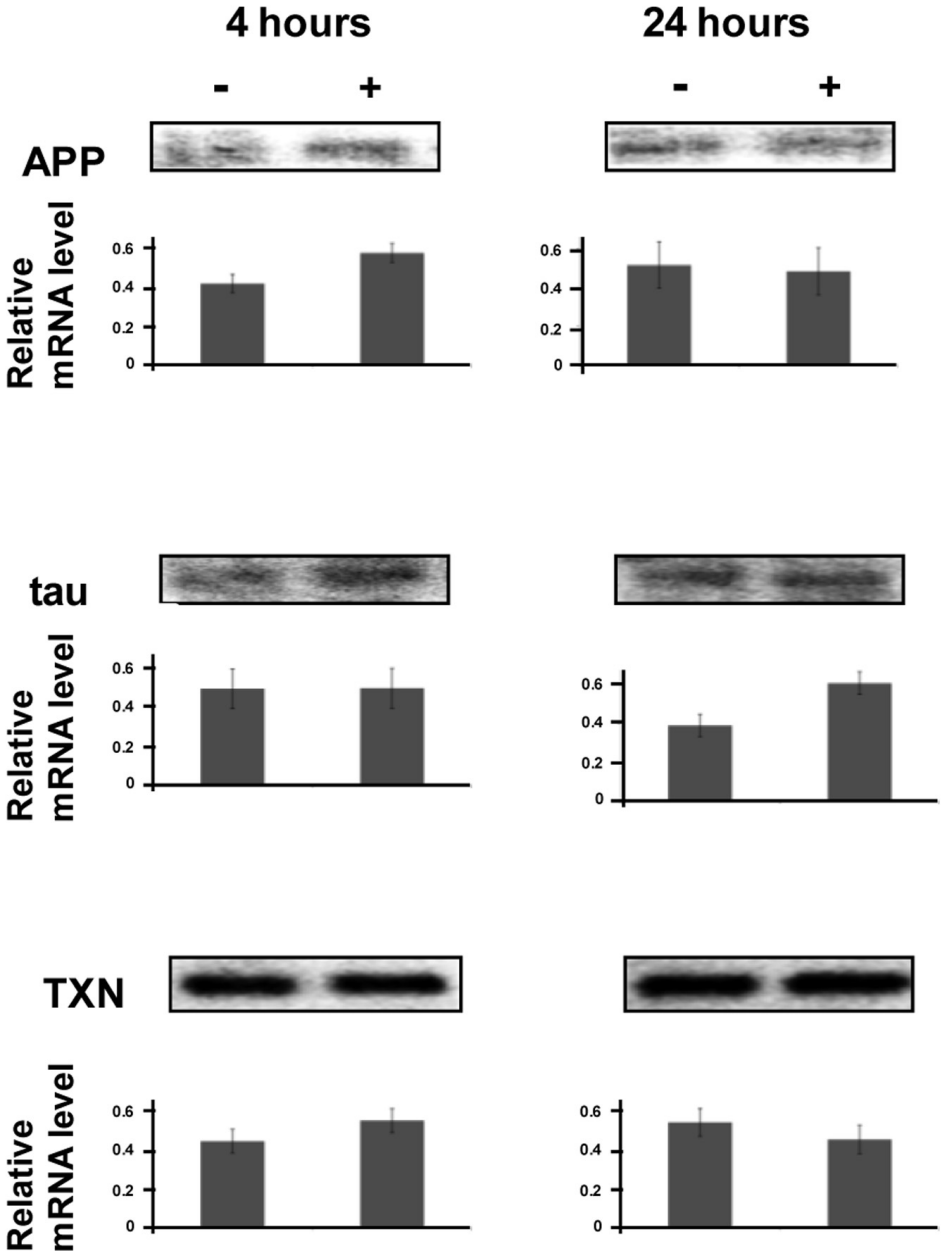
mRNA levels do not account for the change in protein levels following hippuristanol treatment. Northern analysis 4 or 24 hours following 10 minutes 10 µM hippuristanol treatment, using probes specific to APP, TAU, Actin and TXN, indicates no significant decrease in these mRNA levels. Mean relative proportions of RNA with standard errors are indicated in the charts beneath each gel image.

To confirm that the changes in the levels of these proteins were due to translational rather than transcriptional effects, northern analysis of total cellular RNA was performed (figure 3). mRNA levels for APP, tau and TXN did not decrease following hippuristanol treatment for 4 or 24 hours, in parallel with the β-actin control, confirming that hippuristanol was not inhibiting transcription.

### The 5′ UTRs of APP and tau are sufficient to mediate repression in a luciferase reporter model

**Figure 4:**
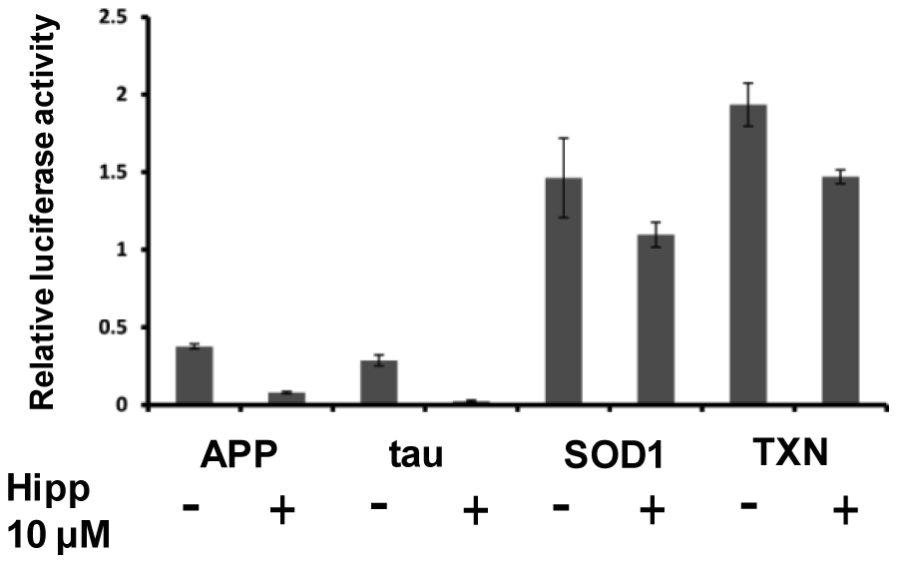
Translational inhibition of APP and tau is mediated via the 5′ UTR. Firefly luciferase reporter plasmids containing the 5′ UTR of APP, tau, SOD1 or TXN upstream of a destabilized firefly luciferase open reading frame were transfected into SH-SY5Y cells +/− 10 µM hippuristanol. The dramatic reduction of luciferase expression in the APP and tau 5′ UTR constructs following hippuristanol treatment is not mirrored by the SOD1 and TXN 5′ UTR containing plasmids. Luciferase activity was normalized to a cotransfected *Renilla* control reporter and means and standard errors of at least three replicates are shown.

Our model predicts that the specific reduction of APP and tau protein levels following eIF4A inhibition depends on features in the 5′ UTRs of these mRNAs. To test this, we cloned the 5′ UTR sequences of APP, tau, SOD1 and TXN upstream of the firefly luciferase open reading frame in pGL4.14 Following co-transfection of these constructs with a control *Renilla* luciferase open reading frame containing a short unstructured 5′ UTR into SH-SY5Y cells we observed that expression from the APP and tau 5′UTR constructs was markedly reduced (to 21% and 8% of controls respectively) following hippuristanol treatment, but the SOD1 and TXN 5′ UTR containing plasmids were relatively resistant to this inhibition (75% and 76% of controls respectively)(figure 4). To eliminate the possibility that cryptic promoter elements within these 5′ UTR sequence could undermine these assays, the cmv promoter in these constructs was deleted by *Ase*I restriction digestion and religation, which completely ablated luciferase activity (data not shown).

### eIF4A inhibition increases cell proliferation in response to oxidative stress

**Figure 5:**
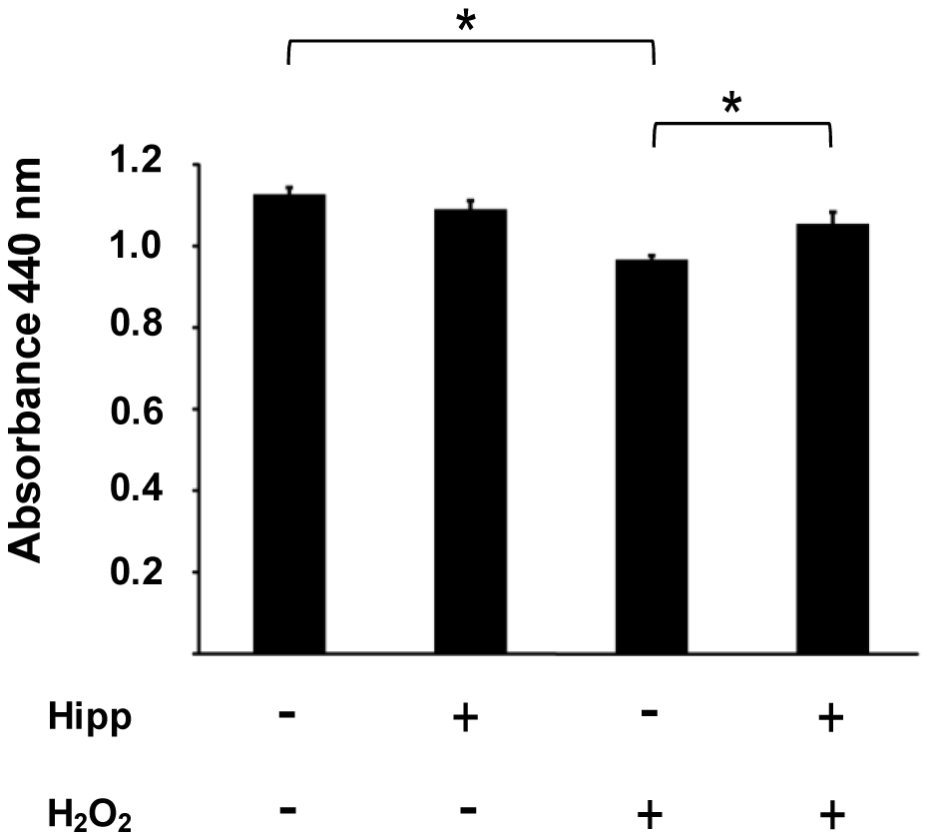
Hippuristanol treatment protects cells from oxidative stress. SH-SY5Y cells were pre-treated with hippuristanol or DMSO control for 4 hours, followed by 50 µM H_2_O_2_ or control treatment for two hours. After a 22 hour recovery period, viability was determined by WST assay. Significant differences (p≤0.05 by t-test) are indicated by asterisks.

SH-SY5Y cells were treated with hydrogen peroxide to induce oxidative stress. Upon treatment of control cells with hydrogen peroxide, there is a small but reproducibly significant reduction in viability. However, treatment with 10 µM hippuristanol confers significantly higher viability upon cells undergoing oxidative stress compared with those in which eIF4A is uninhibited (figure 5).

## Discussion

We have demonstrated that a small molecule inhibitor of protein synthesis can specifically down-regulate APP and tau protein levels in cultured cells of neurological origin. In addition, secreted Aβ levels are also reduced. In contrast, the polysomal association and protein levels of control genes predicted to have neuroprotective functions are resistant to this inhibition. Despite vast amounts of research, current treatment for Alzheimer's disease relies on alleviating symptoms rather than reversing disease progression. The search for novel approaches to AD therapy is, therefore, as important as ever.

Upon treatment with hippuristanol, mRNAs encoding APP and tau become associated with fewer ribosomes, indicating a reduction in their translation. Eukaryotic translation is regulated primarily at initiation, in which a complex of translation factors assembles at the 5′ end of an mRNA molecule to recruit the translational machinery [25]. 5′ UTRs of mRNAs vary significantly in their length, and in their potential to form secondary structure [26]. Increased 5′ UTR length and structure represents a barrier to the scanning ribosomal subunit, and increases the requirement for eIF4A, a DEAD-box RNA helicase which forms part of the initiation complex [27], and is the most abundant translation initiation factor. To confirm that the 5′ UTRs of APP and tau are particularly dependent on eIF4A we used a luciferase reporter system in which candidate 5′ UTRs are positioned upstream of a luciferase open reading frame.

The requirement for eIF4A in experimental translation systems has been shown to be proportional to 5′ UTR structure [27], and the small number of mRNAs that have been shown to be regulated by eIF4A contain long structured 5′ UTRs [19], [28], [29], [30], [31]. Additionally, experiments involving the APP 5′ UTR upstream of a reporter open reading frame showed an inverse correlation between 5′ UTR structure and reporter expression [20]. Analysis of mammalian mRNA structure has determined that messages predicted to be “difficult” to translate due to increased length, structure and other regulatory features in the 5′ UTR tend to encode regulatory rather than housekeeping proteins, and it is this subset of mRNAs that will be particularly reliant on eIF4A for efficient expression [32]. Consistent with this, hippuristanol appears to be well tolerated by cultured cells at the concentrations used, and has a specific and reversible effect on translation [21].

We therefore envisage a model in which inhibition of eIF4A reduces the expression of a subset of regulatory and stress response genes, while leaving housekeeping functions unaffected. The increased polysomal association of TXN, SOD1 and NDUFB2 indicates that RNA-RNA interactions necessary for optimal translation may be stabilized in the absence of active eIF4A.

eIF4A may have a particular role in neuronal translation, as suggested by the restriction of expression of BC1/BC200 RNA to neuronal tissue, where it is thought to interact with and inhibit eIF4A [33]. Patterns of BC200 expression are markedly different in the brains of people with Alzheimer's disease in comparison with those of the healthy elderly, although whether this is a response to AD or a contributing factor is unclear [34]. Additionally, HuD, which is essential for neuronal development, has recently been found to interact with eIF4A [35]. Two almost identical, and so far functionally indistinguishable, isoforms of eIF4A exist in mammalian cells: eIF4AI and eIF4AII. eIF4AI expression is upregulated during retinoic acid induced neural differentiation of P19 cells indicating a specific role in translation of neuronal mRNAs [36]. eIF4AII has been implicated in neural patterning where it has been proposed to up-regulate a subset of mRNAs based on their 5′ UTR structure [37]. Data from the human protein atlas suggest that eIF4AII is more highly expressed in both glial and neuronal cells than eIF4AI, a pattern also reflected in malignant gliomas [38]. Additionally, the eIF4AI/II paralogue, eIF4AIII, which is localized to the nucleus in most cell types and not usually thought to be involved in translation, is found to be associated with (cytoplasmic) mRNAs in somatodendritic regions of neurons [39]. eIF4AIII in these cells has been linked to increased mRNA decay, and eIF4AIII knockdown is associated with an increase in protein synthesis and increased synaptic strength. Interestingly, eIF4AIII has been shown to be inhibitory to eIF4AI/II dependent translation *in vitro* [40]. We therefore propose a model in which 5′ UTR length and structure increase the sensitivity of APP and tau mRNAs to eIF4A inhibition, whereas housekeeping mRNAs and at least a subset of mRNAs predicted to be neuroprotective are resistant to this inhibition.

It is known that the mRNAs of tau and APP contain elements in their 5′ UTRs that regulate their translation, including IRESes [17], [18], and it is plausible that these confer an increased requirement for eIF4A helicase activity to facilitate 40S ribosomal subunit scanning. We have recently demonstrated such a requirement for eIF4A for the cellular IRESes of n-myc, l-myc and c-myc mRNAs [19]. Additionally, at 194 and 320 nts respectively, the 5′ UTRs of APP (NM_000484) and tau (NM_016835) are long in comparison to those of SOD1, TXN and NDUFB2 (148, 63 and 64 nts respectively; NM_000454, NM_003329, NM_004546).

Other modulators of translation initiation are also likely to be important in regulating gene expression in AD, in particular the eIF2alpha kinases PKR and PERK, which can, like hippuristanol, regulate translation initiation. PKR and PERK are two stress induced eIF2alpha kinases which inhibit global translation rates by phosphorylating the translation initiation factor eIF2alpha, preventing eIF2alpha recycling. Active PKR (and inhibited eIF2alpha) are increased in AD models and in patients' brains, and correlate with decreased cognitive ability [41]. PERK is also increased in AD neurons, where it is associated with an increase in aberrant tau protein accumulation [42], and Aβ production [43]. Interestingly, the PI3 kinase inhibitors wortmannin and LY294002 have been proposed to decrease secreted Aβ in a high-throughput cell based assay, with wortmannin showing similar effects in a mouse AD model [44]. Although no strong conclusions were drawn as to the mechanism responsible for this Aβ reduction, it is consistent with inhibition of translation via the mTOR pathway.

SOD1 (superoxide dismutase-1) is responsible for metabolizing superoxide radicals, and hence protecting cells from oxidative stress, a proposed causative factor in AD. Overexpression of SOD1 in neuronal cells protects against the toxic effects of Aβ in cultured neuronal cells [45], [46]. In mice models of AD, increased SOD1 expression ameliorates the cerebrovascular toxicity associated with APP overexpression [23]. TXN (thioredoxin) is also involved in combating oxidative stress and has been shown to have neuroprotective roles [24]. TXN expression is lower in the brains of AD sufferers than in unaffected individuals. Moreover, in cultured neuronal cells, TXN is oxidized by Aβ indicating a role in defence against Aβ mediated oxidative stress. Overexpression of TXN in cultured neuronal cells can protect against the toxic effects of Aβ [47]. NDUFB2 (NADH-ubiquinone oxidoreductase 1 beta subcomplex, 2) is encoded in the nucleus, but is part of the multisubunit mitochondrial NADH:ubiquinone oxidoreductase (complex I), which has been identified as a factor linking oxidative stress and aging, including cognitive aging [22], [48]. Interestingly, complex I proteins have been found to be deregulated in triple transgenic AD mice (expressing mutant tau, APP and PS1) which exhibit both Aβ and tau pathologies [16]. In addition, NDUFB2 is known to be upregulated during ischemic shock, and is proposed to have a role in regulating cellular oxidant levels [49].

The simultaneous inhibition of APP and tau translation by eIF4A inhibition, in concert with increased expression of proteins protective against oxidative stress could therefore represent a new approach for AD intervention. It is well established that oxidative stress occurs early in AD pathology, and that neuronal cells are particularly susceptible to oxidative damage. We have demonstrated that hippuristanol treatment protects against hydrogen peroxide treatment in cultured cells, indicating an additional neuroprotective role for eIF4A inhibitors. We propose that the nature of the 5′ UTRs of the mRNAs tested determines their response to eIF4A inhibition: the mRNAs encoding APP and tau contain long structured 5′ UTRs, in contrast to those of TXN, SOD1 and NDUFB2. Moreover, APP and tau 5′ UTRs contain IRES elements, allowing cap-independent translation initiation [17], [18]. Although little is known of the translation factor requirements for cellular IRESes, we have recently shown that the myc family of IRESes requires eIF4A for proper function [19]. We can therefore speculate that this may also be the case for the APP and tau IRESes, and this is currently under investigation. We propose that housekeeping genes, which tend to have short, unstructured 5′ UTRs are largely unaffected by the concentration of hippuristanol used. However, it is more difficult to propose an explanation for the increase in synthesis of the neuroprotective proteins tested. We can speculate that RNA structure elements (or RNA∶RNA or RNA∶protein interactions) in the 5′ UTR is required for optimal translation, and that these are stabilized by the absence of eIF4A helicase activity, but this remains to be tested. The involvement of HuD – eIF4A interactions in control of neuronal genes indicates that interactions of eIF4A with other proteins may be important [35]. Nevertheless it is clear that eIF4A inhibition has differential effects on the synthesis of proteins involved in Alzheimer's disease, and that these effects could represent a novel approach to AD intervention.

## Materials and Methods

### Cell culture

Two 15cm plates of cultured cells (SH-SY5Y, N2a or HeLa) were used per treatment. Cells were grown to 70% confluency then treated with 10 µM hippuristanol (a kind gift from Prof Ya-Ching Chen, National Taiwan University), or DMSO control for 10 minutes, at which point translation elongation was arrested by the addition of cycloheximide (1 mg/ml) on ice. Cells were lysed (300 mM NaCl, 15 mM MgCl_2_, 15 mM Tris-HCl pH 7.5, 0.1 mg ml^−1^ cycloheximide, 1 mg ml^−1^ heparin, 1% triton X-100) then cytoplasmic extract was loaded onto 10%–50% sucrose gradients (300 mM NaCl, 15 mM MgCl_2_, 15 mM Tris-HCl pH 7.5, 0.1 mg ml^−1^ cycloheximide, 1 mg ml^−1^ heparin) and centrifuged at 38,000 rpm for two hours. OD260nm absorbance was determined with simultaneous 1 ml fraction collection. Oxidative stress assay: SH-SY5Y cells were pre-treated with 10 µM hippuristanol for 4 hours. Cells were washed twice with PBS then replaced with media, 10 µM hippuristanol, 50µM H_2_O_2_ (or controls) and incubated at 37°C for 2hours. After this time treatment media was removed, cells were washed twice and fresh media only was added to each well. After a 22 hour recovery period, 10µl WST-1 (Roche, Mannheim, Germany) was added to the 90µl recovery media in each well and cell viability measured after 45 minutes following manufacturer's instructions.

### Analysis of mRNA and protein levels

Northern analysis was performed on the gradient fractions to determine the positions of mRNAs for β-actin, PABP, APP, tau, SOD1, NDUFB2 and TXN, using probes derived from PCR products to the coding regions of these genes. Northern analysis of polysome gradients was quantified using Image Quant (GE Healthcare), expressing the value for each gradient fraction as a percentage of the total for the whole gradient. Each experiment was performed as at least three independent replicates and a typical example shown. Northern blots of total cellular RNA were quantified using the gel analysis function of ImageJ [50] and values for each protein corrected for loading errors by comparison with actin controls, and expressed as a fraction of paired control (DMSO) and experimental (10 µM hippuristanol) samples. Means and standard errors of at least three replicates of each experiment are shown. Western analysis was performed on lysates from SH-SY5Y cells treated with 10 µM hippuristanol (+) or DMSO control (−) for 4 or 24 hours with antibodies specific to APP, Aβ, tau, TXN, and β-actin (Primary antibodies used in this study were all sourced from Cell Signaling Technology, Beverly, MA, USA: anti-APP (2452); anti-ß-Amyloid (2454); anti-tau (4019); anti-TXN 1 (2285). Secreted Aβ was measured by western analysis of SH-SY5Y cell culture media. 500µl of medium was taken from each well of a 12 well plate 24 hours after treatment. Medium was then concentrated by microcon column (Millipore, Billerica, MA, USA) to a final volume of 20µl. Four independent biological replicates were conducted per treatment. A 70 kDa band was detected by the APP specific antibody representing a cleavage product specific to K+ isoforms [14]. The 56 kDa product is interpreted as an aggregate of Aβ, as has previously been reported for western analysis of brain tissue [51]. Western blots were quantified using the gel analysis function of ImageJ [50], and values for each protein corrected for loading errors by comparison with tubulin and actin controls, and expressed as a fraction of paired control (DMSO) and experimental (10 µM hippuristanol) samples. Means and standard errors of at least three replicates of each experiment are shown. Significance was determined by t-test, a p value≤0.05 indicated by an asterisk. Immunoprecipitation was performed following ^35^S-methionine labelling using the above antibodies, as described in [52]. Immunoprecipitation experiments were quantified using the gel analysis function of ImageJ [50] and expressed as a fraction of paired control (DMSO) and experimental (10 µM hippuristanol) samples. Means and standard errors of at least three replicates of each experiment are shown. Significance was determined by t-test, a p value≤0.05 indicated by an asterisk. ELISAs were performed using a kit designed to detect human Aβ 1–42 (reference KHB3441, Invitrogen, Paisley, UK), following manufacturer's instructions. SH-SY5Y medium for ELISAs was collected 24 hours following treatment with 10 µM hippuristanol or DMSO control, before addition of protease inhibitors (EDTA free, Roche, Basel, Switzerland, plus 25mM NaF) and glycerol phosphate (50 mM).

### Luciferase reporter assays

Firefly luciferase reporter plasmids were constructed by inserting the cmv promoter from pGL3.1 (Promega, Madison, WI), amplified using primers (CMV F, CTGGCCGGTACCTCGCGATGTACGGGCCAGAT and CMV R, CTCGAGAAGCTTAAGTTTAAACGCTAGCCAGC), and inserted between the KpnI and *Hin*dIII sites of pGL4.15 and pGL4.80 to create pGL4.15cmv and pGL4.80cmv respectively. A cloning intermediate, php15, was created by inserting part of the human ODC 5′ UTR (accession NM_002539) amplified using primers (ODCHP F, GATTACAAAGCTTCTCGAGGGGCGAATACGAATTCGTCA and ODCHP R, GATTACAAAGCTTTTAATTAAGGATCCGTCTTCCCGCCGCC) into the *Hin*dIII site of pGL4.15cmv. TXN and SOD1 5′ UTRs were amplified from SH-SY5Y cDNA using primers (TXN F GATACACTCGAGTTTGGTGCTTTGGATCCATT, TXN R GATACATTAATTAACTTGGCTGCTGGAGTCTGAC, SOD1 F GATACACTCGAGGTTTGGGGCCAGAGTGGGCG, SOD1 R GATACATTAATTAAAACTCGCTAGGCCACGCCGA), and match nucleotides 1–63 of NM_003329 and 1–148 of NM_000454 respectively. APP and tau 5′ UTRs were synthesised by Genscript (Piscataway, NJ), to match nucleotides 1–194 of NM_201414 and 1–320 of NM_001123066 respectively, flanked by *Xho*I and *Pac*I restriction sites. Following restriction digestion with *Xho*I and *Pac*I, these APP, tau, SOD1 and TXN 5′ UTR sequences were inserted between the *Xho*I and *Pac*I sites of php15 to generate pGL4.15cmvAPP5, pGL4.15cmvtau5, pGL4.15cmvSOD1.5 and pGL4.15cmvTXN5 respectively. 800 ng each of the pGL4.15cmv[5′ UTR] plasmids and control pGL4.80cmv plasmid was co-transfected into each well of a 24 well plate containing sub-confluent SH-SY5Y cells. Following recovery for 4 hours, the cells were split into a 96 well plate, and allowed a further 4 hours recovery. 10 µM hippuristanol or DMSO control was added, and cells harvested after 4 hours. Luciferase levels were assayed using the Dual Luciferase Assay (Promega, Madison, WI) following manufacturer's instructions.
